# Associations of Thyroid Hormones Profile During Normal Pregnancy and Postpartum With Anxiety, Depression, and Obsessive/Compulsive Disorder Scores in Euthyroid Women

**DOI:** 10.3389/fnins.2021.663348

**Published:** 2021-08-04

**Authors:** Panagiota Konstantakou, Nikos Chalarakis, Georgios Valsamakis, Evangelos Grigoriou Sakkas, Eleni Vousoura, Alexandros Gryparis, Grigorios Evangelou Sakkas, George Papadimitriou, Ioannis Zervas, George Mastorakos

**Affiliations:** ^1^Unit of Endocrinology, Diabetes Mellitus and Metabolism, Medical School, Aretaieion Hospital, National and Kapodistrian University of Athens, Athens, Greece; ^2^Unit of Endocrinology, Diabetes Mellitus and Metabolism, Alexandra General Hospital, Athens, Greece; ^3^Department of Psychiatry, Eginition Hospital, Athens University Medical School, Athens, Greece; ^4^Rea Maternity, Private Hospital, Athens, Greece

**Keywords:** thyroid hormone, pregnancy, postpartum, anxiety, depression, obsessive—compulsive disorder, psychometrics

## Abstract

**Objective:**

Thyroid dysfunction (overt and subclinical) has been consistently linked to pregnancy adversity and abnormal fetal growth and development. Mood disorders such as anxiety, depression, and obsessive-compulsive disorder (OCD) are frequently diagnosed during pregnancy and at postpartum, and emerging evidence suggests association with impaired offspring neurodevelopment and growth. This study aimed to examine potential associations between thyroid function and mood symptoms during pregnancy and postpartum.

**Design:**

This is a prospective study measuring thyroid hormones and assessing mood symptoms by employing specific questionnaires in the same cohort of 93 healthy pregnant women at the 24th (2nd trimester) and 36th (3rd trimester) gestational weeks and at the 1st postpartum week.

**Methods:**

Serum thyroid hormones, TSH, anti-TPO, and anti-Tg antibodies were measured at the 24th (2nd trimester) and 36th (3rd trimester) gestational weeks and at the 1st postpartum week. Specific validated questionnaires were employed at the same time-points to assess separately symptoms of anxiety [Generalized Anxiety Disorder Inventory (GADI), Penn State Worry Questionnaire (PSWQ), STAI-State Anxiety inventory (STAI-S), STAI-Trait Anxiety Inventory (STAI-T)], depression [Edinburgh Postnatal Depression Scale (EPDS), Stein’s Blues Scale (BLUES), Beck Depression Inventory (BDI)], and obsessive compulsive disorder (OCD) [Yale-Brown Obsessive Compulsive scale (Y-BOCS)].

**Results:**

At the 2nd trimester, GADI score correlated negatively with FT3 (*p* < 0.010, *r* = −0.545) and positively with TSH (*p* < 0.050, *r* = 0.837) concentrations; GADI, PSWQ, EPDS and Y-BOCS scores correlated negatively with FT4 concentrations (*p* < 0.010, *r* = −0.768; *p* < 0.010, *r* = −0.384; *p* < 0.050, *r* = −0.364; *p* < 0.010, *r* = −0.544, respectively). At the 3rd trimester, BLUES score correlated positively with rT3 concentrations (*p* = 0.00, *r* = 0.89); GADI, EPDS, and Y-BOCS scores correlated negatively with FT4 concentrations (*p* = 0.001, *r* = − 0.468; *p* = 0.036, *r* = −0.39; *p* = 0.001, *r* = −0.625, respectively); GADI, STAI-S, and Y-BOCS scores correlated positively with TSH concentrations (*p* = 0.015, *r* = 0.435; *p* = 0.024, *r* = 0.409 *p* = 0.041, *r* = 0.389, respectively). At postpartum, PSWQ, STAI-T, EPDS, and BDI scores correlated positively with rT3 concentrations (*p* = 0.024, *r* = 0.478; *p* = 0.014, *r* = 0.527; *p* = 0.046, *r* = 0.44; *p* = 0.021, *r* = 0.556, respectively, Y-BOCS score correlated positively with TSH (*p* = 0.045, *r* = 0.43), and BLUES score correlated positively with anti-TPO antibody concentrations (*p* = 0.070, *r* = 0.586).

**Conclusion:**

The reported findings demonstrate positive associations between low-normal thyroid function at the 2nd and 3rd trimesters of pregnancy and postpartum with anxiety, depression, and OCD scores.

## Introduction

Mood disorders are common during pregnancy and postpartum and manifest as mild to severe depression, anxiety and obsessive-compulsive disorder (OCD), in previously healthy women (O’Hara and Swain Annette, 1996; Kemp et al., 2003; Gaynes et al., 2005). According to the majority of the studies, during pregnancy and at postpartum, depression affects approximately 10–25% and 10–15% of women, respectively, while anxiety affects 5.6–15% and 6.1–27.9% of women, respectively (Andersson et al., 2003; Wenzel et al., 2005; Faisal-Cury and Rossi Menezes, 2007; Vesga-López et al., 2008; Rubertsson et al., 2014; Dennis et al., 2017). Although mood disorders (mainly depression) appear to be more frequent at these time periods, approximately half of the cases remain undiagnosed (Bonari et al., 2004; Seehusen et al., 2005; Ban et al., 2012). A meta-analysis reported that women during pregnancy and postpartum had a 45% (matched risk ratio, mean = 1.45) and a 138% (matched risk ratio, mean = 2.38) increased risk of developing OCD, respectively (Russel et al., 2013).

Homeostasis of thyroid hormones is challenged during pregnancy due to adaptive changes of the hypothalamic-pituitary thyroid (HPT) axis. In most pregnant women, the steep rise in serum concentrations of maternal thyroid-binding globulin, which follows the increase in estrogen concentrations, results in increased serum total triiodothyronine (TT3) and total tetraiodothyronine (TT4) concentrations. Free T3 (FT3) and free T4 (FT4) concentrations typically decrease after the 1st trimester, usually remaining within low-normal range (Glinoer, 1997; Soldin et al., 2004). At postpartum, TT4 concentrations decline for approximately 6 weeks before plateauing at pre-pregnancy concentrations (Pedersen et al., 1993). Recently, in a cohort of euthyroid pregnant women, we have shown that TT4 and reverse T3 (rT3) mean concentrations at the 1st postpartum week were significantly lower than those at the 2nd and the 3rd trimesters (Sakkas et al., 2018). Thyrotropin hormone (TSH) concentrations decrease during the 1st trimester of pregnancy following the stimulatory effect of placental human chorionic gonadotropin on TSH receptor, while during the 2nd and 3rd trimesters, they rise again to reach pre-pregnancy concentrations and usually remain stable throughout pregnancy (Glinoer, 1997).

In non-pregnant population, several reports have stressed the comorbidity of thyroid dysfunction and mood disorders: overt and subclinical hyperthyroidism have been associated with anxiety (Trzepacz et al., 1988; Sait Gönen et al., 2004), mania (Burch and Messervy, 1978), and depression (Trzepacz et al., 1988; Hage and Azar, 2012) while overt and subclinical hypothyroidism have been associated with depressive (Gulseren et al., 2006; Demartini et al., 2010) and anxiety clinical characteristics (Burch and Messervy, 1978; Monzani et al., 1993; Sait Gönen et al., 2004; Thomsen et al., 2005; Gulseren et al., 2006; Demartini et al., 2010; Hage and Azar, 2012; Ittermann et al., 2015). Data on the association of thyroid function with mood disorders during pregnancy and postpartum are insufficient. Studies assessing anxiety separately from depression (often comorbid) are missing. A clear association of anxiety with thyroid function has not been reported. At the 3rd trimester of pregnancy, low-normal FT4 concentrations were associated with depression (Pedersen et al., 2007) while, at postpartum, data on the association between thyroid hormones and depression are equivocal (Ijuin et al., 1998; Kent et al., 1999; Bloch et al., 2003; Albacar et al., 2010; Sylvén et al., 2013). Evidence of an association of thyroid function with OCD during pregnancy and postpartum is missing as yet.

This study investigated prospectively the possible associations between thyroid function and symptoms of anxiety, depression, and OCD (all symptoms studied separately by employing specific, validated questionnaires) at the 2nd and 3rd trimesters of pregnancy and postpartum in the same cohort of healthy pregnant women.

## Subjects and Methods

### Subjects

In an outpatient clinic of a University Obstetrics and Gynecology department, 121 primiparous Caucasian pregnant women were informed consecutively about this study and 105 of them accepted to participate by giving their informed consent. Patients were enrolled and followed up prospectively during the 2nd and 3rd trimesters of pregnancy and at postpartum (from 2015 to 2017). Inclusion criteria are as follows: clinical and biochemical euthyroidism according to TSH and FT4 concentrations (normalcy within 0.6–3.4 mIU/L and 10.3–15.5 nmol/L, respectively), normal weight, or slightly overweight. Exclusion criteria are as follows: past or present history of diagnosed psychosis, depression, anxiety, and OCD based on medical history and screened with the Symptom Checklist (SCL-90-R) (Donias et al., 1991); autoimmune thyroid disease based on thyroid antibodies concentrations (Sakkas et al., 2018); pre-existing autoimmune disorder and treatment for hepatitis C or AIDS; insulin-dependent diabetes; and twin pregnancy. None of the women were on thyroid hormone replacement, while all were euthyroid. Regarding potential confounders for the presence of thyroid antibodies, given that all women came from the same geographical area, they were considered to have been exposed to similar levels of iodine and pollution (Shukla et al., 2018). Selenium levels were not measured in these women. Regarding other potential factors/confounders (apart from thyroid function) that may affect the incidence of anxiety, depression, or OCD, all women were primiparous and had similar young age whereas none was a current smoker or had a medical history of mental illness. Socioeconomic status and educational level were not assessed (Rubertsson et al., 2014). Finally, after application of the exclusion criteria, 93 pregnant women aged 33.2 ± *2*.*4* years old (mean ± SD), with normal weight or overweight, BMI 24.5 ± 1.1 kg/m^2^ (mean ± SD), were included in this study. The study has been approved by the ethics committee of our Institution, and all study participants gave their written informed consent.

### Protocol

All participants were seen in the outpatient clinic at the 2nd and 3rd trimesters of their pregnancy (24th and 36th week of gestation, respectively) and at the 1st postpartum week. At each visit, they underwent biochemical and psychometric tests. Biochemical evaluation was performed on serum obtained after an overnight fast. Blood samples were drawn at 8:00 a.m. for measurement of TT3, FT3, rT3, TT4, FT4, TSH, anti-thyroid peroxidase (anti-TPO), and anti-thyroglobulin (anti-Tg) antibodies and were stored immediately at −75°C for analysis. Psychometric evaluations were conducted by the same skilled psychiatrist (N.C.) with standardized structured instruments (Generalized Anxiety Disorder Inventory (GADI); Penn State Worry Questionnaire (PSWQ); STAI-State Anxiety inventory (STAI-S); STAI-Trait Anxiety Inventory (STAI-T); Edinburgh Postnatal Depression Scale (EPDS); Stein-BLUES scale (BLUES); Beck’s Depression Inventory (BDI); Yale-Brown Obsessive Compulsive scale (Y-BOCS)]. There were no dropouts during the time period of this study.

### Hormone Assays

Serum TT3, FT3, rT3, TT4, FT4, TSH, anti-TPO, and anti-Tg antibody concentrations were measured by employing automated chemiluminescence immunoassay (Immulite 2000, Siemens Gwynned LL55 4EL, United Kingdom). For TT3, FT3, TT4, FT4, TSH, anti-TPO, and anti-Tg antibodies, the intra- and interassay coefficients of variation (CV) and the analytical sensitivity (AS) were, respectively, 5.5%, 7%, and 0.19 ng/ml; 9.1%, 9.4%, and 1.50 pmol/L; 4.6%, 6%, and 0.3 μg/dL; 5.9%, 6.4%, and 3.00 pmol/L; 3.8%, 4.6%, and 0.004 μUI/ml; 5.2%, 7.2%, and 10 U/ml; and 4.9%, 5.7%, and 20 U/L. Reverse T3 was measured using a radioimmunoassay (RIA, rT3 RADIM, ROMA, Italy) with intra- and interassay CV and AS at 8.54%, 6.21%, and 0.009 ng/ml, respectively. High and low concentrations were defined according to reference limits.

### Psychometric Questionnaires

#### The Symptom Checklist-90-Revised (SCL-90-R)

This is a widely used, 90-item self-report questionnaire assessing a broad range of psychological and psychopathology symptoms (Donias et al., 1991). Each item is scored on a 5-point Likert scale of distress ranging from 0 (none) to 4 (extreme). Greater scores indicate greater prevalence of symptom occurrence during the time reference. This questionnaire assesses nine primary symptom dimensions, including depression, anxiety, obsessive-compulsive symptoms, psychoticism, and somatization, as well as other general indices of global distress. No total range or cut-off score is reported in this questionnaire. Responses are scored and normed, comparing the individual with the same-age cohort.

#### Generalized Anxiety Disorder Inventory (GADI)

This is a self-report, 18-item, questionnaire employed to assess cognitive, somatic, and sleep symptoms in the context of generalized anxiety and panic disorder (Argyropoulos et al., 2007). Items are scored from 0 (“not at all”) to 4 (“extremely”) and total scores range from 0 to 72 with greater scores indicating greater symptom severity over the past 2 weeks.

#### Penn State Worry Questionnaire (PSWQ)

This is a self-report, 16-item questionnaire employed to assess the trait of worry (Lialiou et al., 2011). Items are rated on a Likert scale from 1 (“not at all typical of me”) to 5 (“very typical of me”), and total scores range from 16 to 80. Greater PSWQ scores signify greater levels of pathological worry. A cut-off score of 50 is commonly used to define probable clinical levels of anxiety.

#### State and Trait Anxiety Inventory (STAI)

This questionnaire consists of two separate 20-item questionnaires, one assessing state anxiety and the other trait anxiety (Fountoulakis et al., 2006b). In the State questionnaire, respondents rate how they feel “right now,” while in the Trait questionnaire they rate how they feel “generally.” Items are scored on a 4-point Likert scale, from 1 (“almost never”) to 4 (“almost always”). For each questionnaire, scores range from 20 to 80. Greater scores suggest greater state or trait anxiety. A cut-off score of 40 is commonly used to define probable clinical levels of anxiety.

#### Edinburgh Postnatal Depression Scale (EPDS)

This is a widely employed, 10-item questionnaire assessing symptoms of depression and anxiety during perinatal period. Scores range from 0 to 80. Scores of 12 and greater are indicative of clinically significant symptoms of perinatal depression (Leonardou et al., 2009).

##### Stein’s Blues Scale

This is a self-rating scale with 13 items which evaluates maternal blues. Scores range from 0 to 26, with greater scores denoting greater severity of maternal blues, while 8 is the clinical cut-off score, above which significant mood swings are suspected (Yamashita, 1994).

#### Beck Depression Inventory (BDI)

This is a widely used, 21-item, self-report measure of depression (Lykouras et al., 1998). BDI items are ranked on a 4-point scale (0–3). Cut-off scores are as follows: (i) 0–9 minimal depression, (ii) 10–18 mild depression, (iii) 19–29 moderate depression, and (iv) 30–63 severe depression.

#### The Yale-Brown Obsessive Compulsive Scale (Y-BOCS)

This is a self-report version of the Y-BOCS employed to assess obsessions and compulsions (Goodman et al., 1989). It consists of 10 items on a 5-point Likert scale from 0 (“no symptoms”) to 4 (“severe symptoms”). Total scores range from 0 to 40. It is considered the gold standard for the assessment of OCD pathology. Scores under 7 are considered non-clinical, scores from 8 to 15 reflect mild severity, from 16 to 23 moderate severity, while symptoms over 24 reflect severe cases of OCD.

### Statistical Analysis

The normality of the distributions was checked both graphically (i.e., using histograms and q-q plots) and formally (i.e., using Kolmogorov-Smirnov test). Normally distributed variables are presented as mean ± SD, while non-normally as median (25th and 75th percentiles). All hormonal variables were normally distributed except for anti-TPO and anti-Tg antibodies. The quantitative results of the employed psychometric questionnaires exhibited non-normal distribution. To test the change of each variable during pregnancy, analysis of variance (ANOVA) and the non-parametric Wilcoxon tests were employed for variables with normal and non-normal distributions, respectively. To test the changes of hormonal and psychometric variables over the three time points of the study protocol during pregnancy and postpartum, ANOVA for repeated measures was employed. The previously reported TSH, FT4, FT3, TT4, TT3, rT3, anti-TPO, and anti-Tg antibody concentrations measured in peripheral maternal blood in the studied subjects at the three time points of the study protocol were employed in this study for correlation analyses according to authorization received from *Clinical Endocrinology* (Sakkas et al., 2018). To test for associations between variables, the Spearman correlation analysis was employed. Longitudinal bivariate models using fixed effects were performed with time taken into consideration. Random effects were examined and were not found to be statistically significant. Therefore, these effects were not included in the models. Statistical significance was set at *p* < 0.05. *P*-values were calculated by employing the SPSS statistical package (SPSS).

## Results

### Baseline Hormonal and Psychometric Variables at the 2nd and 3rd Trimesters of Pregnancy and Postpartum

Serum TT3, TT4, FT3, FT4, TSH, anti-TPO, and anti-Tg antibodies mean concentrations at the 2nd and 3rd trimesters of pregnancy and postpartum and their differences at these three time points are reported in Table 1. STAI-S questionnaire scores increased significantly (*p* = 0.042) from the 2nd to the 3d trimester of pregnancy. The rest of the applied questionnaires did not change significantly during pregnancy and postpartum. None of the participants developed a psychiatric illness during pregnancy although some women have scored in certain questionnaires above the respective cut-off points. Following assessment with the psychometric questionnaires during the 2nd and 3rd trimesters and at postpartum 27.8% (*n* = 25), 25.6% (*n* = 23), and 14.4% (*n* = 13) of the studied women, respectively, were considered possible candidates for anxiety while 11.1% (*n* = 10), 7.8% (*n* = 7), and 5.6% (*n* = 5) of the studied women, respectively, were considered possible candidates for depression.

**TABLE 1:** Hormonal and psychometric variables at pregnancy and postpartum.

*N* = 93	2nd trimester	3rd trimester	Postpartum
TT4 (μg/dl)	11.87 ± 0.26	11.59 ± 0.33	9.45 ± 0.65*^#^
FT4 (pmol/l)	8.37 ± 0.76	8.93 ± 0.92	12.99 ± 1.35*^#^
TT3 (ng/ml)	1.75 ± 0.05	1.67 ± 0.05	1.29 ± 0.06*^#^
FT3 (pmol/l)	4.93 ± 0.24	5.10 ± 0.23	4.95 ± 0.20
rT3 (ng/ml)	0.35 ± 0.01	0.38 ± 0.01	0.30 ± 0.02*^#^
TSH (μU/ml)	1.33 ± 0.1	1.55 ± 0.16	1.08 ± 0.15
anti-TPO (UI/ml) median (25th–75th)	10.0 (4.70–10.2)	10.42 (5.02–11.6)	12.473*^#^ (7.043–14.40)
anti-Tg (UI/ml) median (25th–75th)	19.00 (17.44–21.53)	18.90 (16.93–23.03)	19.37 (17.87–22.48)
GADI median (25th–75th)	20.00 (11.75–30.25)	17.00 (13.00–23.00)	14.00 (7.00–25.00)
PSWQ median (25th–75th)	44.00 (37.50–53.00)	43.00 (38.00–49.00)	41.00 (38.00–50.00)
STAI-S median (25th–75th)	37.50 (32.00–37.50)	40.00 (33.00–50.00)*	37.00 (27.00–47.75)
STAI-T median (25th–75th)	38.00 (34.05–43.00)	35.50 (30.75–44.00)	34.00 (25.75–41.25)
EPDS median (25th–75th)	7.00 (4.00–11.00)	7.00 (5.00–10.00)	6.50 (3.00–11.00)
BLUES median (25th–75th)	14.50 (9.00–16.00)	13.00 (11.00–19.00)	10.00 (6.25–16.00)
BDI median (25th–75th)	9.00 (5.00–12.00)	7.00 (5.00–11.00)	7.50 (4.25–11.75)
Y-BOCS median (25th–75th)	13.00 (10.00–16.00)	12.00 (10.00–14.75)	11.00 (8.00–14.50)

Normally distributed variables are presented as mean ± SD, while non-normally distributed variables as median (25th and 75th percentiles). The asterisk (*) indicates statistically significant difference from the 2nd trimester; the pound sign (#) indicates statistically significant difference from the 3rd trimester. Serum TT3, TT4, FT3, FT4, TSH, anti-TPO and anti-Tg antibodies mean concentrations during the 2nd and 3rd trimesters of pregnancy and at postpartum and their differences at the three time points of the study have been published before (17).

*GADI, Generalized Anxiety Disorder Inventory; PSWQ, Penn State Worry Questionnaire; STAI- S, State Anxiety Inventory; STAI- T, Trait Anxiety Inventory; EPDS, Edinburgh Postnatal Depression Scale; BLUES, Stein’s Maternal Blues Scale; BDI, Beck Depression Inventory; Y-BOCS, Yale- Brown Obsessive Compulsive Scale.*

### Correlations Among Psychometric and Hormonal Variables at the 2nd Trimester of Pregnancy

Significant correlations among hormonal and psychometric variables at the 2nd trimester are presented in Table 2. GADI score correlated negatively with FT3 concentrations (*p* < 0.010, *r* = −0.545); GADI, EPDS, PSWQ and Y-BOCS scores correlated negatively with FT4 concentrations (*p* < 0.010, *r* = −0.768; *p* < 0.050, *r* = −0.364; *p* < 0.01, *r* = −0.384; *p* < 0.010, *r* = −0.544 respectively); GADI score correlated positively with TSH concentrations (*p* < 0.050, *r* = 0.837).

**TABLE 2:** Significant correlations among hormonal and psychometric variables at the 2nd and 3rd trimesters and post-partum.

	2nd trimester			3rd trimester			Postpartum		
	FT3	FT4	TSH	rT3	FT4	TSH	rT3	TSH	anti-TPO
GADI	*r* = −0.545	*r* = −0.768	*r* = 0.837		*r* = −0.468	*r* = 0.435			
	*p* < 0.010	*p* < 0.010	*p* < 0.05		*p* = 0.018	*p* = 0.015			
PSWQ		*r* = −0.384					*r* = 0.478		
		*p* < 0.010					*p* = 0.024		
STAI-S						*r* = 0.409			
						*p* = 0.024			
STAI-T							*r* = 0.527		
							*p* = 0.014		
EPDS		*r* = −0.364			*r* = −0.39		*r* = 0.44		
		p < 0.050			*p* = 0.036		*p* = 0.046		
BLUES				*r* = 0.89					*r* = 0.586
				*p* = 0.000					*p* = 0.070
BDI							*r* = 0.556		
							*p* = 0.021		
Y-BOCS		*r* = −0.544			*r* = −0.625	*r* = 0.389		*r* = 0.43	
		*p* < 0.010			*p* = 0.001	*p* = 0.041		*p* = 0.045	

*GADI, Generalized Anxiety Disorder Inventory; PSWQ, Penn State Worry Questionnaire; STAI- S, State Anxiety Inventory; STAI- T, Trait Anxiety Inventory; EPDS, Edinburgh Postnatal Depression Scale; BLUES, Stein’s Maternal Blues Scale; BDI, Beck Depression Inventory; Y-BOCS, Yale- Brown Obsessive Compulsive Scale.*

### Correlations Among Psychometric and Hormonal Variables at the 3rd Trimester of Pregnancy

Significant correlations among hormonal and psychometric variables at the 3rd trimester are presented in Table 2. BLUES score correlated positively with rT3 concentrations (*p* = 0.000, *r* = 0.89); GADI, EPDS, and Y-BOCS scores correlated negatively with FT4 concentrations (*p* = 0.010, *r* = −0.468; *p* = 0.036, *r* = −0.39; *p* = 0.001, and *r* = −0.625, respectively); GADI, STAI-S, and Y-BOCS scores correlated positively with TSH concentrations (*p* = 0.015, *r* = 0.435; *p* = 0.024, *r* = 0.409; *p* = 0.041, and *r* = 0.389, respectively).

### Correlations Among Psychometric and Hormonal Variables at Postpartum

Significant correlations among hormonal and psychometric variables at postpartum are presented in Table 2. PSWQ, STAI-T, EPDS, and BDI scores correlated positively with rT3 concentrations (*p* = 0.024, *r* = 0.478; *p* = 0.014, *r* = 0.527; *p* = 0.046, *r* = 0.44; *p* = 0.021, *r* = 0.556, respectively); BLUES score correlated positively with anti-TPO antibody concentrations (*p* = 0.07, *r* = 0.586); Y-BOCS score correlated positively with TSH concentrations (*p* = 0.045, *r* = 0.43).

### Correlations Among Psychometric and Hormonal Variables Over the 2nd and 3rd Trimesters of Pregnancy and Postpartum

Longitudinal bivariate models showed a significant positive change of GADI, EPDS, and YBOCS scores (taken separately as dependent variables) with time and TSH taken together as independent variables (*p* = 0.006, *p* = 0.058, and *p* = 0.020, respectively) as presented in Table 3. Longitudinal bivariate models showed a significant negative change of GADI, EPDS, and YBOCS scores (taken separately as dependent variables) with time and FT4 taken together as independent variables (*p* < 0.001, *p* = 0.031, and *p* < 0.001, respectively).

**TABLE 3:** Results from longitudinal bivariate models for GADI, EPDS, and YBOCS as dependent variables with time, TSH, and FT4 as independent variables.

	Coef.	SE	*t*	95%CI
	**Dependent variable = GADI**			
TSH	4.303	1.508	2.853^∗∗^	1.30–7.31
FT4	–1.211	0.176	–6.862^∗∗∗^	−1.56—0.86
	**Dependent variable = EPDS**			
TSH	1.598	0.829	1.928	−0.059–3.255
FT4	–0.248	0.112	−2.219^∗^	−0.472—0.024
	**Dependent variable = YBOCS**			
TSH	1.292	0.532	2.428^∗^	0.217–2.366
FT4	–0.301	0.066	–4.545^∗∗∗^	−0.436—0.165

*p < 0.05, *⁣*p < 0.01, *⁣*⁣*p < 0.001.

*GADI, Generalized Anxiety Disorder Inventory; PSWQ, Penn State Worry Questionnaire; STAI- S, State Anxiety Inventory; STAI- T, Trait Anxiety Inventory; EPDS, Edinburgh Postnatal Depression Scale; BLUES, Stein’s Maternal Blues Scale; BDI, Beck Depression Inventory; Y-BOCS, Yale- Brown Obsessive Compulsive Scale.*

## Discussion

We found that at the 2nd and 3rd trimesters of pregnancy, anxiety scores (GADI, PSWQ, STAI-S), in these euthyroid women without history of mental illness, correlated negatively with circulating FT3 and FT4 and positively with TSH concentrations, while at postpartum anxiety scores (PSWQ, STAI-T) correlated positively with rT3 concentrations. These findings indicate a positive association between low-normal thyroid function with anxiety scoring during pregnancy and postpartum. In the past, the majority of studies in pregnancy and postpartum assessed concurrently depressive mood and anxiety with regard to thyroid function (Kuijpens et al., 2001; Pop et al., 2006; Falah-Hassani et al., 2017). In men and non-pregnant women, anxiety disorders are more often encountered in patients suffering from thyroid disorders than in healthy controls (Placidi et al., 1998; Thomsen et al., 2005; Sareen et al., 2006). Many studies conducted in non-pregnant states reported a positive association between either overt (Thomsen et al., 2005; Panicker et al., 2009; Ittermann et al., 2015) or subclinical (Monzani et al., 1993) hypothyroidism or autoimmune stigmata of thyroiditis (positive anti-TPO antibodies) with anxiety (Monzani et al., 1993; Carta et al., 2004), whereas others did not (Engum et al., 2002, 2005; Eaton et al., 2010). Recently, Thvilum et al. (2014) reported a significantly increased prevalence of a neuropsychiatric diagnosis, including anxiety, before diagnosis of hypothyroidism. In the same study, individuals diagnosed with hypothyroidism presented an excessive risk for subsequent diagnosis of anxiety. Of note, anxiety symptoms improve with achievement of euthyroidism after treatment of either overt or subclinical hypothyroidism (Gulseren et al., 2006). Anxiety, irritability, and emotional instability seen in thyroid disease may reflect β-adrenergic dysfunction and over-activity in CNS (Mills et al., 1986; Stern et al., 1995). Older studies reported increased urinary norepinephrine (NE) excretion and significantly elevated plasma NE concentrations in hypothyroid patients (Christensen, 1972; Coulombe et al., 1977). This apparent contradiction with the clinical signs of hypothyroidism which rather imply decreased peripheral sympathetic tone, might be due to peripheral desensitization to adrenergic stimulation (Polikar et al., 1990a, b). The involvement of determinants of thyroid hormone action within CNS, such as specific thyroid hormone transporters [mono-carboxylate transporter 8 (MCT8)] and deiodinase enzymes could also explain the reported clinical association between anxiety symptoms and hypothyroidism (Venero et al., 2005; Wallis et al., 2008).

In this study, at the 2nd and 3rd trimesters of pregnancy, depression scores (EPDS) correlated negatively with circulating FT4 concentrations, while at the 3rd trimester depression scores (BLUES) correlated positively with rT3 concentrations. At postpartum, depression scores (EPDS, BDI) correlated positively with rT3 concentrations while scores derived from the BLUES questionnaire showed a trend of positive correlation with anti-TPO concentrations. These findings indicate a positive association between low-normal thyroid function with depression scoring during pregnancy and postpartum. In the past, in the 3rd trimester of pregnancy, low-normal FT4 concentrations have been associated positively with depressive mood (Pedersen et al., 2007). At postpartum, some authors reported absence of any correlation between thyroid hormones and depression although others found a positive association of subclinical hypothyroidism and postpartum depressive mood (Ijuin et al., 1998; Kent et al., 1999; Bloch et al., 2003; Albacar et al., 2010; Sylvén et al., 2013). For decades, depressive mood disorder and HPT axis physiology are under investigation in the general population with contradictory and inconclusive reports (Fountoulakis et al., 2006a). Hypothyroidism may affect mood possibly *via* decreased central serotonin (5-HT) activity. Thyroid hormones modulate the serotonergic system while plasma 5-HT concentrations correlate positively with thyroid hormone concentrations (Cleare et al., 1995, 1996). Low-normal thyroid function is associated with poorer treatment response and lower remission rate in depressive patients (Abulseoud et al., 2007, 2013). However, increased FT4 plasma concentrations (even within normalcy) were described in depressive disorders (Forman-Hoffman and Philibert, 2006; Berent et al., 2014; Medici et al., 2014). It is not yet clear, whether the abnormal concentrations of the thyroid hormones are the cause or consequence of depression (Delitala et al., 2016).

Finally, in the present study, at the 2nd and 3d trimesters of pregnancy, OCD scores (Y-BOCS) correlated negatively with circulating FT4 concentrations while at the 3rd trimester and at post-partum, they correlated positively with TSH concentrations. To the best of our knowledge, this is the first report indicating a positive association between low-normal thyroid function with OCD scoring during pregnancy and postpartum. A meta-analysis revealed that women during pregnancy and postpartum had a 45 and a 138% increased risk, respectively, for developing OCD (Russel et al., 2013). The latter is part of the DSM-5 obsessive-compulsive spectrum disorders; its course follows a chronic fluctuating pattern related to stressful life events; its main symptoms are intrusive thoughts (obsessions) and ritualistic behavior (compulsions) (Stewart et al., 2004; American Psychiatric Association, 2013). Similarly to depression, 5-HT neurotransmission is implicated in OCD pathophysiology (Henley et al., 1991; Sandrini et al., 1991). The confirmed interaction of thyroid hormones physiology with the serotonergic system together with the fact that thyroid hypofunction is associated with decreased central 5-HT activity (Cleare et al., 1995, 1996) could explain the negative correlation of OCD questionnaire scores with the thyroid hormones concentrations during pregnancy and postpartum found in this study.

The simultaneous psychometric evaluation of anxiety, depression, and OCD in the same cohort of euthyroid pregnant women without history of mental illness at the 2nd and 3rd trimester of pregnancy as well as at postpartum is reported for the first time, to the best of our knowledge. The prospective design of the study and the separate rating of anxiety, depression, and OCD add to the consistency of the reported data. Limitations of this study are as follows: the studied women may not be representative of the general population because they were recruited from a tertiary care university hospital where a higher number of high risk pregnancies are attended; the lack of psychometric measurements during the 1st trimester of pregnancy; and the number of the studied women is relatively small.

In this study, measures for depression, anxiety, and OCD improved in figures but not in a statistically significant way in the course of pregnancy and postpartum. Regarding anxiety, according to a published meta-analysis in 221,974 women from 34 countries, when gold standard diagnostic interviews were employed, the rates dropped from 18% in the 1st trimester to 15 and 15% in the 2nd and 3rd trimesters, respectively. Prevalence continued to drop at postpartum and ranged from 9.3 to 9.9% across 1st year (Dennis et al., 2017). The reported data indicate independent positive associations between low-normal thyroid function with anxiety, depression, and OCD scores by the applied multiple specific psychometric questionnaires which add to the strength of the present study. Indeed, by longitudinal bivariate models, a significant positive change of GADI, EPDS, and YBOCS scores (taken separately as dependent variables) with time and TSH (taken together as independent variables) was observed, while a significant negative change of these scores with time and FT4 (taken together as independent variables) was observed. The consistency of these associations is suggestive of a pathophysiologic link. Of note, many of the limbic system structures, where thyroid hormone receptors are prevalent, have been implicated in the pathogenesis of mood disorders (Ruel et al., 1985; Bradley et al., 1992).

Depressive and anxiety disorders, during pregnancy and at postpartum, are complex clinical conditions with atypical features and symptoms, overlapping with “physiologic” mood changes. Maternal prenatal anxiety has been related to increased postnatal risk of neurodevelopmental disorders (separately and independently of depression) (O’Connor et al., 2002; Sheridan et al., 2008; Glover, 2015; Tiemeier, 2017). Fetuses of prenatally anxious mothers showed lower weight increase from the 2nd trimester to delivery as well as lower neonatal weight, length, and ponderal indexes (Pinto et al., 2017). Epigenetic mechanisms, including fetal hypothalamic-pituitary-adrenal axis dysregulation, among others, have been proposed as mediators of the effect of prenatal anxiety on fetal and neonatal growth (Wadhwa et al., 2004; Hompes et al., 2013; Valsamakis et al., 2017). On the other hand, recent meta-analyses provide evidence that prenatal maternal depression is associated with preterm birth (Grote et al., 2010; Grigoriadis et al., 2013) and low birth weight (Grote et al., 2010; Jarde et al., 2016), while fetal exposure to maternal depression is a risk factor for childhood psychopathology (O’Donnell et al., 2013; Sandman et al., 2015). The present study demonstrates negative correlations between thyroid function and the evaluation of mood disorders by questionnaires during pregnancy and at postpartum. In future studies, thyroid hormone concentrations in mid- and late pregnancy and postpartum could be employed (alone or combined with other markers) to identify women at risk for developing mood disorders at pregnancy and postpartum.

## Data Availability Statement

The raw data supporting the conclusions of this article will be made available by the authors, without undue reservation.

## Ethics Statement

The studies involving human participants were reviewed and approved by the Ethics Committee of Aretaieio University Hospital, National and Kapodistrian University of Athens. The patients/participants provided their written informed consent to participate in this study.

## Author Contributions

All authors listed have made a substantial, direct and intellectual contribution to the work, and approved it for publication.

## Conflict of Interest

The authors declare that the research was conducted in the absence of any commercial or financial relationships that could be construed as a potential conflict of interest. The reviewer ST declared a past co-authorship with one of the authors GM to the handling editor.

## Publisher’s Note

All claims expressed in this article are solely those of the authors and do not necessarily represent those of their affiliated organizations, or those of the publisher, the editors and the reviewers. Any product that may be evaluated in this article, or claim that may be made by its manufacturer, is not guaranteed or endorsed by the publisher.
